# Centenarians in nursing homes during the COVID-19 pandemic

**DOI:** 10.18632/aging.202743

**Published:** 2021-03-02

**Authors:** Anne-Laure Couderc, Florian Correard, Emilie Nouguerède, Julie Berbis, Dominique Rey, Aurélie Daumas, Patrick Villani

**Affiliations:** 1 Internal Medicine, Geriatry and Therapeutic Unit, AP-HM, Marseille, France; 2 Aix-Marseille University, CNRS, EFS, ADES, Marseille, France; 3 Pharmacy Department, AP-HM, Marseille, France; 4 Aix-Marseille University, Marseille, France; 5 Public Health Department, AP-HM, Marseille, France

**Keywords:** centenarians, COVID-19, nursing home, mortality, symptoms

## Abstract

Background: Centenarians are known to be successful agers compared to other older adults.Objective: The objective of the present study was to compare coronavirus disease (COVID-19) symptoms and outcomes in centenarians and other residents living in nursing homes.

Design-Setting-Subjects-Methods: A retrospective multicenter cohort study was conducted using data from 15 nursing homes in the Marseille area. Older residents with confirmed COVID-19 between March and June 2020 were enrolled. The clinical and biological characteristics, the treatment measures, and the outcomes in residents living in these nursing homes were collected from the medical records.

Results: A total of 321 residents were diagnosed with COVID-19 including 12 centenarians. The median age was 101 years in centenarians and 89 years in other residents. The most common symptoms were asthenia and fever. Three centenarians (25%) experienced a worsening of pre-existing depression (vs. 5.5% of younger residents; *p* = 0.032). Mortality was significantly higher in centenarians than in younger residents (50% vs. 21.3%, respectively; *p* = 0.031). A quarter of the younger residents and only one centenarian were hospitalized. However, 33.3% of the centenarians received treatment within the context of home hospitalization.

Conclusion: Worsening of pre-existing depression seems to be more frequent in centenarians with COVID-19 in nursing homes. This population had a higher mortality rate but a lower hospitalization rate than younger residents.

## INTRODUCTION

Since December 2019, there has been a growing number of patients worldwide infected with the novel coronavirus 2019 (SARS-CoV-2). The recent literature confirms that older adults appear to be more susceptible to the virus [1], as well as more likely to present the most severe cases, in particular those with comorbidities (hypertension, diabetes, cancer, cardiological and respiratory diseases) [2] and/or frailties such as functional or cognitive impairments [3]. Among older adults, the number of centenarians has increased over the past 20 years. In France, there were 20 944 centenarians in 2017 [4]. Their susceptibility to the disease is different from that of other older people. Previous surveys have shown that thirty percent of centenarians are not affected by major age-related diseases including dementia. Others have described a phenomenon of “compression of morbidity” (including cancer) in centenarians leading them to spend a relatively short period of their lives with age-related diseases or disabilities [5, 6]. Genomic and genetic studies carried out in this particular population have described several mechanisms underlying this exceptional longevity [7].

We conducted a search in PubMed for articles on screening and cases of severe acute respiratory syndrome coronavirus 2 (SARS-CoV-2) in centenarians published between January 1 and November 10, 2020. We identified three studies that had included centenarians with COVID-19 [8–10] and three others that had included patients over 90 years [11–13]. None described specific characteristics and outcomes in centenarians managed for COVID-19 in nursing homes.

The objectives of this study carried out by the geriatric and therapeutic department at Marseille University Hospital (AP-HM) were (1) to describe and (2) to compare comorbidities, clinical and biological characteristics, treatment measures, and outcomes in centenarians and other residents diagnosed with COVID-19 in 15 nursing homes in the Marseille area.

## RESULTS

In our study, 321 residents were diagnosed with COVID-19 including 12 centenarians. The median age in centenarians was 101 years (interquartile range (IQR) 100-102.75) and 11 out of 12 were women. The median age of the other residents was 89 years (IQR 82-93) and 70.2% were women. Irrespective of the age group, the most frequent comorbidities were cognitive impairment, depression, and hypertension. Centenarians had chronic renal disease more often than younger residents (33.3% vs. 12.5%) but none had chronic pulmonary disease. The demographic, clinical, and geriatric characteristics and comorbidities in centenarians and younger residents are detailed in Table 1. Even though the differences were not statistically significant, centenarians were more likely to be malnourished according to the body mass index (BMI) than younger residents at the time of COVID-19 infection (58.3% vs. 43.4%) and to have impaired autonomy (75% belonged to an iso-resource group ≤ 3 vs. 69% of younger residents). Concerning chronic medications, only four centenarians (33.3%) were exposed to polypharmacy compared to 74.8% of younger residents (p = 0.004).

**Table 1:** Demographic, geriatric, and comorbid characteristics of centenarians vs residents aged 65-99 years.

**Characteristics**	**Patient 65 to 99 years old (n=309)**		**Patient 100 years and over (n=12)**		**p-value**
	**n or median**	**% or IQR**	**n or median**	**% or IQR**	
**Age**	*89*	*82-93*	*101*	*100-102.75*	
<80	57	18.4	-	-	
80-89	123	39.8	-	-	
90-99	129	41.8	-	-	
100-101	-		8	66.7	
102-105	-		4	33.3	
**Gender**					
Men	92	29.8	1	8.3	
Women	217	70.2	11	91.7	**<0.0001**
**Vaccination**					
Flu	237	76.7	9	75.0	1.000
Pneumococcus	133	43.0	4	33.3	0.567
**Comorbidities**					
Cognitive impairment*	229	74.1	10	83.3	0.737
Depression	154	49.8	7	58.3	0.770
History of psychiatric illness	22	7.1	-	-	
Hypertension	166	53.7	6	50.0	1.000
Coronaropathy	44	14.2	-	-	
Chronic obstructive pulmonary disease (COPD)	28	9.1	-	-	
Asthma	7	2.3	-	-	
Respiratory deficiency	11	3.6	-	-	
Atrial fibrillation	66	21.4	3	25.0	0.725
Cardiac deficiency	49	15.9	2	16.7	1.000
Dyslipidemia	21	6.8	-	-	
Diabetes	48	15.5	1	8.3	0.701
Cerebrovascular disease	59	19.1	2	16.7	1.000
Cancer history	18	5.8	2	16.7	0.167
Renal chronic disease	40	12.5	4	33.3	0.067
**Number of comorbidities**	*4*	*3-6*	*4.5*	*3.25-6*	1.000
0	4	1.3	0	0.0	
1-4	162	52.4	6	50.0	
5-7	142	46.0	6	50.0	
**Iso-resource group**					0.744
1	60	19.4	4	33.3	
2	154	49.8	5	41.8	
3 and more	47	30.4	2	16.6	
unknown	1	0.4	1	8.3	
Nutritional status (BMI) **	*22.3*	*19.3-26.1*	*20*	*18.1-22.8*	
BMI <21 kg/m^2^	134	43.4	7	58.3	0.142
BMI <18 kg/m^2^	32	10.4	2	16.7	0.628
BMI ≥ 30 kg/m^2^	34	11.0	1	8.3	1.000
**Polypharmacy** (≥5 drugs)	231	74.8	4	33.3	**0.004**
Antidepressant	52	16.8	4	33.3	0.234
Psychotic Drug	59	19.1	1	8.3	0.704

*Cognitive impairment: Mini Mental State Examination score <24 and/or presence of cognitive symptoms and/or cognitive disorders history.

** Malnutrition was defined as body mass index < 21 kg/m^2^, severe malnutrition as body mass index < 18 kg/m^2^ and obesity as body mass index ≥30 kg/m^2^.

BMI= body mass index; n= number; IQR=interquartile range.

Table 2 describes COVID-19 related symptoms, biology, and treatment among the centenarians and the other residents. The most common symptoms were asthenia (50% of centenarians and 51.2% of younger residents) and fever (50% of centenarians and 42.4% of younger residents). Anorexia was more common among centenarians (41.7%) than among the others (21.4%) but, unlike the younger residents, the centenarians did not suffer from headaches, myalgia, or falls. Three centenarians (25%) experienced a worsening of pre-existing depression (vs. 5.5% of younger residents; *p* = 0.032).

**Table 2:** COVID-19 related symptoms, biology, treatment, and clinical outcomes of centenarians vs residents aged 65-99 years.

**Characteristics**	**Patient 65 to 99 years old (n=309)**		**Patient 100 and over (n=12)**		**p-value**
	**n**	**%**	**n**	**%**	
**Asymptomatic**	61	19.7	2	16.7	1.000
**Symptoms**					
Dyspnea	115	37.2	4	33.3	1.000
Dry cough	84	27.2	2	16.7	0.526
Fever	131	42.4	6	50.0	0.768
Hypothermia	15	4.9	1	8.3	0.465
Asthenia	159	51.5	6	50.0	1.000
Anorexia	66	21.4	5	41.7	0.147
Gastrointestinal signs	56	18.1	2	16.7	1.000
Ear, Nose, Throat (ENT)	25	8.1	2	16.7	0.267
Headache	7	2.3	-	-	
Myalgia	9	2.9	-	-	
Delirium	42	13.6	2	16.7	0.673
Worsening of depression	17	5.5	3	25.0	**0.032**
Altered consciousness	18	5.8	1	8.3	0.525
Fall	26	8.4	-	-	
Biological characteristics*					
Anemia	90	29.1	6	50.0	0.194
Neutropenia	16^a^	6.6	1^α^	10.0	0.508
Lymphopenia	69^a^	28.4	4^α^	40.0	0.480
Thrombopenia	21^a^	8.6	1^α^	10.0	0.604
C-reactive protein>10mg/L	163^b^	71.8	7^β^	77.8	1.000
**Treatment**					
Antibiotics**	201	65.0	10	83.3	0.231
Azithromycin	231	74.8	10	83.3	0.737
Hydroxychloroquine	99	32.0	1	8.3	0.113
Hydration	150	48.5	9	75.0	0.084
Preventive anticoagulation	85	27.5	4	33.3	0.743
Oxygen therapy	116	37.5	5	41.7	0.769
**Drug-drug interactions**	139	45.0	5	41.7	1.000
**Adverse event**	33	10.7	1	8.3	1.000
**Hospitalization**	76	24.6	1	8.3	0.306
In COVID unit	74	23.9	1	8.3	0.307
**Home hospitalization**	111	35.9	4	33.3	1.000
**Palliative care**	42	13.6	4	33.3	0.77
**Death**	66	21.3	6	50.0	**0.031**
**Death due to COVID**	45	84.9	6	100.0	0.583
**Place of death**					
COVID unit	27	8.7	1	8.3	0.296
Nursing home	39	12.6	5	41.7	0.062

*Anemia was defined as hemoglobin level <120 g/L; Neutropenia as neutrophils level <1.5 G/L; Lymphopenia as lymphocytes level <1.0 G/L; Thrombopenia was defined as platelets level < 150 G/L.

**Antibiotics used were penicillin, ceftriaxone, macrolides (other than azithromycin) and tetracycline.

^a^n=66 values were missing; ^α^n=2 values were missing; b 82 missing values; β 3 missing values.

Ten centenarians received azithromycin, one received hydroxychloroquine, and 4 received preventive anticoagulation. None received antiviral drugs. Moreover, 5 centenarians had oxygen therapy and 9 received intravenous or subcutaneous hydration. Regarding supportive care, 4 centenarians were given analgesics and/or anxiolytics. Drug-drug interactions were detected between azithromycin and psychotic drugs and/or antidepressants for five centenarians. COVID-19 care was comparable among the younger residents even if they received hydroxychloroquine more often (32%) and hydration (48.5%) less often.

Hospitalization concerned 24.6% of younger residents and only one centenarian (to manage dyspnea). Four centenarians received palliative care, perfusions for hydration, and hydroxychloroquine treatment initiation within the context of home hospitalization. Finally, 50% of centenarians (n = 6) and 21.3% of younger residents died from COVID-19 during hospitalization or in nursing homes (*p* = 0.031) and centenarians were more likely to die in nursing homes (Table 2).

The comparison of the 12 centenarians showed that three out of the six deceased centenarians only had one COVID-19-related symptom (fever, cough, or dyspnea). Lymphopenia was more prevalent in deceased centenarians than surviving centenarians. Centenarians who died from COVID-19 were more likely to have hypertension, diabetes, obesity or severe malnutrition (according to BMI), and severe disability (according to the iso-resource group) than those who survived (Table 3). Deceased centenarians received more oxygen therapy than survivors and conversely, surviving centenarians had more preventive anticoagulation.

**Table 3:** Characteristics of the 12 centenarians.

**Characteristics**	**Survivors** **(n=6)**	**Deceased** **(n=6)**
**Age** (years)		
100	3	2
101-105	3	4
**Gender**		
Men	0	1
Women	6	5
**Flu Vaccination**	3	6
**Comorbidities**		
Cognitive impairment *	5	5
Hypertension	2	4
Atrial fibrillation	2	1
Cardiac deficiency	2	0
Diabetes	0	1
Cerebrovascular disease	2	0
Cancer history	0	2
Renal chronic disease	2	2
Depression	4	3
**Iso-resource group** (n=11)		
1	1	3
2 and more	4	3
**Nutritional status** (n=9) **		
BMI <21 kg/m^2^	4	3
BMI <18 kg/m^2^	0	2
BMI ≥30 kg/m^2^	0	1
**Polypharmacy** (≥ 5 drugs)	3	1
**Asymptomatic**	2	0
**Symptoms**		
Dyspnea	2	2
Dry cough	1	1
Fever	3	3
Hypothermia	0	1
Asthenia	4	2
Anorexia	2	3
Gastrointestinal signs	1	1
Ear, Nose, Throat (ENT)	2	0
Delirium	1	1
Depression worsening	1	2
**Treatment**		
Antibiotics ***	4	6
Azithromycin	5	5
Hydroxychloroquine	0	1
Hydration	4	5
Preventive anticoagulation	3	1
Oxygen therapy	1	4
**Drug-drug interactions**	3	2
**Adverse event**	0	1
**Biological characteristics** (n=9) ****		
Anemia	3	3
Neutropenia	0	1
Lymphopenia	1	3
C-reactive protein >10mg/l	4	3

*Cognitive impairment: Mini Mental State Examination score <24 and/or presence of cognitive symptoms and/or cognitive disorders history.

**Malnutrition was defined as body mass index < 21 kg/m^2^, severe malnutrition as body mass index < 18 kg/m^2^ and obesity as body mass index ≥30 kg/m^2^.

***Antibiotics used were penicillin, ceftriaxone, macrolides (other than azithromycin) and tetracycline.

****Anemia was defined as hemoglobin level <120 g/L; Neutropenia as neutrophils level <1.5 G/L; Lymphopenia as lymphocytes level <1.0 G/L; thrombopenia was defined as platelets level < 150 G: L / BMI= body mass index; N= number.

A complementary comparative analysis dividing the population into four age groups (65-79, 80-89; 90-99 and >100 years old) suggests that anorexia increased with age (p = 0.036) and that, compared to other groups, centenarians were mostly treated with a combination of antibiotics (p = 0.024) and were more often offered hydration (p < 0.001). Moreover, the death rate increased with age (p = 0.029). The worsening of depression was not significantly different between the four age groups but was nevertheless more prevalent in the centenarian age group (25% vs. 7.0%, 4.1%, 6.2% in the 65-79, 80-89, and 90-99 age groups, respectively) (Supplementary Table 1).

## DISCUSSION

To our knowledge, this is the first multicenter study to describe and compare the clinical, geriatric, and biological characteristics and outcomes in centenarians and other residents with COVID-19 in nursing homes.

The centenarians studied had a poorer survival rate than the other residents but had a low hospitalization rate, as home hospitalization was favored.

In our study, the mortality rate was 50% among the 12 centenarians, which is higher than among the other residents (24.6%). In the literature, the mortality rate of COVID-19 for hospitalized older adults varies from 14.8% to 31% [13, 14] but little is known about the COVID-19-related mortality rate in nursing homes. On June 1, 2020, the COVID-19-related death rate was 27.4% in France [15] and up to 40% in nursing homes in the USA [16]*.* A study of centenarians’ in Lombardy (comparing community-dwelling patients with nursing home residents) showed that COVID-19-related mortality was high for the oldest old adults [17]. A majority of the centenarians who died in our study received home hospitalization at the nursing home. Currently, the hospitalization of centenarians is avoided whenever possible [18]. The hospitalization rate in centenarians varies between 18% and 50% in the literature [18, 19] and the increased number of care home beds has been associated with fewer deaths of older residents in hospital [20]. Moreover, the centenarians who received home hospitalization in our survey had a severe form of COVID-19 and their poor clinical state could contra-indicate hospitalization. This could explain the low hospitalization rate in centenarians. With the COVID-19 pandemic, older persons, and in particular very old persons or those with severe comorbidities, are at particular risk of severe infection and poor outcomes [2, 21]. Despite successful aging, centenarians have a higher mortality rate than younger residents. Nevertheless, there is no concrete evidence that the low hospitalization rate in our study could actually be linked to the higher mortality rate of the centenarian group compared to the younger residents, as our study sample was too small to draw any conclusions. The mortality rate of the centenarians in our study shows the importance of implementing effective COVID-19 prevention measures, especially for nursing home residents.

Concerning demographics and comorbidities, in the centenarian group [22] 91.7% were women compared with only 70.2% in the 65 to 99 years age group, which is in accordance with the literature [23, 24]. Little is known regarding the great longevity of women centenarians but it is thought that it could be more related to environmental conditions than genetic factors [24, 25]. All the more, centenarians had fewer cardiovascular and depression comorbidities than other residents [25], and polypharmacy was less frequent in centenarians (*p* = 0.004) than in other residents in our survey, which is consistent with previous results in older patients living in nursing homes [26]. However, centenarians in nursing homes were more dependent according to the iso-resource group classification and more often suffered from malnutrition than other residents.

Concerning symptoms, centenarians had a fever and asthenia in 50 % of cases, followed by anorexia (41.7%) and dyspnea (33.3%). Anorexia was more frequent in centenarians than in other residents. In the literature, asthenia was particularly frequent in older patients with COVID-19, which is in accordance with our work [2, 13, 27]. Despite our small sample size, the worsening of depression was significantly more prevalent in centenarians than in other residents. Depression has not been described in the literature on older patients with COVID-19, except in a study using the depression score to predict older inpatient mortality [11]. The particularity of this symptom is the increased worry and sadness in residents with pre-existing comorbid depression. However, the worsening of depression can also be associated with asthenia and delirium in older adults. Delirium was present in two centenarians and is commonly under-recognized when superimposed on major neurocognitive disorders, especially during the severe stages of the disease, as it is difficult to find a clear distinction between symptoms attributable to delirium or underlying dementia [28, 29]. Due to the small sample of centenarians and the lack of power of our comparative analysis with the 65 to 99-year-olds, few symptoms were significantly associated with age.

Chronic inflammation (inflammaging) increases with age and is known to play a major role in the physiopathology of COVID-19 [30]. Inflammaging is lower than expected in centenarians, as they develop anti-inflammatory markers that may delay diseases and can partially explain successful aging [31, 32]. The only inflammation marker available in our survey was CRP and we did not find any significant difference in CRP levels according to the age of the residents. However, this is not conclusive, as we collected the biological data retrospectively from the medical files at the nursing homes, and C-reactive protein levels were missing for 26.2% of the residents.

Concerning COVID-19 treatment, a majority of the centenarians received antibiotics, in particular azithromycin, and hydration. Specific treatments (including hydroxychloroquine, antiviral therapy, or immunomodulatory agents) were used less in centenarians than in other residents, which is in accordance with the treatment of hospitalized older patients described in the literature [13, 33].

This study is limited as it was retrospective with a limited number of centenarians. However, the COVID-19 screening policy implemented in French nursing homes involved the systematic screening of all the residents each time a case was detected among staff, health care workers, or residents. Consequently, our sample of residents with symptomatic or asymptomatic coronavirus disease included all COVID-19 cases during the study period. The other strengths of the study concern the detailed collection of clinical, biological, management, and survival data among centenarians with COVID-19 at the time of the pandemic, which is rare in the geriatric literature. The small number of patients did not allow multivariable analysis to highlight factors that may influence deaths or hospitalizations in this population. Moreover, our centenarians with COVID-19 were all living in nursing homes and are more frail than community-dwelling centenarians. However, taking into account that data on centenarians are sparse and that only 305 centenarians were living in the Marseille area in 2017 [4], our small sample was worth describing. We compared their data with other residents and showed differences among symptoms and outcomes. However, our results need to be validated on a larger population from other areas.

Conclusion: Centenarians with COVID-19 in nursing homes had a high mortality rate (50%) but a low hospitalization rate. Home hospitalization was preferred to avoid hospitalizations and allowed patients to receive supportive care. Even in our small sample, clinical differences were observed between centenarians and younger residents regarding COVID-19 symptoms. These specificities should be studied clinically and genetically in larger samples of both nursing home and community-dwelling centenarians to improve detection and management of the disease in this frail population.

## MATERIALS AND METHODS

### Study design and participants

The AP-HM geriatric and therapeutic department invited the 39 nursing homes in the city of Marseille to participate in a thirteen-week retrospective study on centenarians with COVID-19. Fifteen nursing homes answered favorably and nine of them had at least one centenarian with confirmed COVID-19 (detection of SARS-CoV-2 nucleic acid using the real-time reverse transcription-polymerase chain reaction (RT-PCR) assay of nasal swabs) from March 1 to June 2, 2020. None of the residents or their legal representatives refused the use of the medical data. The medical records were collected retrospectively and compared to those of all the younger residents with COVID-19 living in the same nine nursing homes. The clinical outcomes, including hospitalizations and mortality, were monitored up to June 2, which was the final follow-up date. The study was authorized by the National Institute for Health Data (number INDS-MR3109280520) and conducted in accordance with the reference methodology MR-004 approved by the National Commission for Information Technology and Civil Liberties (French CNIL).

### Data collection

Patient medical records in nursing homes were reviewed by trained physicians and researchers working at AP-HM and not involved in the management of the nursing homes. The following data were collected for each patient: demographic and geriatric characteristics (age, gender, nutritional status according to BMI, most recent disability score according to the iso-resource group [residents classified in iso-resource groups ≤ 3 must be assisted in most or all daily activities]), underlying comorbidities, cognitive impairment, chronic medications, polypharmacy (≥ 5 usual drugs per day), and vaccination status.

The date of disease onset was defined as the first day of symptoms or the day of diagnosis with the RT-PCR test for asymptomatic patients. The following symptoms were collected for each patient: general signs (asthenia, anorexia, fever or hypothermia), respiratory signs, ear nose and throat (ENT) signs, myalgia, headache, gastrointestinal signs, worsening of pre-existing depression, and some geriatric syndromes (falls, delirium also known as acute confusional state and altered consciousness [Glasgow coma score < 14]). We also collected the results of laboratory tests and data about COVID-19 management including treatment, notified adverse events, hospitalization, home hospitalization, and palliative care. The causes of death were also recorded.

### Statistical analysis

No statistical sample size calculation was performed, and the sample size was equal to the number of patients enrolled during the study period. Continuous data are presented as the median with the interquartile range (IQR). Categorical variables are summarized by frequency, number, and percentage of residents. Comparative analyses were performed using the Fischer exact test as the centenarian population comprised only 12 patients. Analyses were performed using SPSS software (version 17.0).

## Supplementary Material

Supplementary Table 1

## References

[1] Yang X, Yu Y, Xu J, Shu H, Xia J, Liu H, Wu Y, Zhang L, Yu Z, Fang M, Yu T, Wang Y, Pan S, et al. Clinical course and outcomes of critically ill patients with SARS-CoV-2 pneumonia in Wuhan, China: a single-centered, retrospective, observational study. Lancet Respir Med. 2020; 8:475–81. 10.1016/S2213-2600(20)30079-5 32105632 PMC7102538

[2] Borges do Nascimento IJ, Cacic N, Abdulazeem HM, von Groote TC, Jayarajah U, Weerasekara I, Esfahani MA, Civile VT, Marusic A, Jeroncic A, Carvas Junior N, Pericic TP, Zakarija-Grkovic I, et al. Novel coronavirus infection (COVID-19) in humans: a scoping review and meta-analysis. J Clin Med. 2020; 9:941. 10.3390/jcm9040941 32235486 PMC7230636

[3] Wang H, Li T, Barbarino P, Gauthier S, Brodaty H, Molinuevo JL, Xie H, Sun Y, Yu E, Tang Y, Weidner W, Yu X. Dementia care during COVID-19. Lancet. 2020; 395:1190–91. 10.1016/S0140-6736(20)30755-8 32240625 PMC7146671

[4] POP1B - Population par sexe et âge en 2017 − Commune de Marseille (13055) −Évolution et structure de la population en 2017.. https://www.insee.fr/fr/statistiques/4515319?sommaire=4515349&geo=COM-13055.

[5] Andersen SL, Sebastiani P, Dworkis DA, Feldman L, Perls TT. Health span approximates life span among many supercentenarians: compression of morbidity at the approximate limit of life span. J Gerontol A Biol Sci Med Sci. 2012; 67:395–405. 10.1093/gerona/glr223 22219514 PMC3309876

[6] Ismail K, Nussbaum L, Sebastiani P, Andersen S, Perls T, Barzilai N, Milman S. Compression of morbidity is observed across cohorts with exceptional longevity. J Am Geriatr Soc. 2016; 64:1583–91. 10.1111/jgs.14222 27377170 PMC4988893

[7] Sebastiani P, Solovieff N, Dewan AT, Walsh KM, Puca A, Hartley SW, Melista E, Andersen S, Dworkis DA, Wilk JB, Myers RH, Steinberg MH, Montano M, et al. Genetic signatures of exceptional longevity in humans. PLoS One. 2012; 7:e29848. 10.1371/journal.pone.0029848 22279548 PMC3261167

[8] Tang O, Bigelow BF, Sheikh F, Peters M, Zenilman JM, Bennett R, Katz MJ. Outcomes of nursing home COVID-19 patients by initial symptoms and comorbidity: results of universal testing of 1970 residents. J Am Med Dir Assoc. 2020; 21:1767–73.e1. 10.1016/j.jamda.2020.10.011 33153910 PMC7556822

[9] McMichael TM, Currie DW, Clark S, Pogosjans S, Kay M, Schwartz NG, Lewis J, Baer A, Kawakami V, Lukoff MD, Ferro J, Brostrom-Smith C, Rea TD, et al, and Public Health–Seattle and King County, EvergreenHealth, and CDC COVID-19 Investigation Team. Epidemiology of covid-19 in a long-term care facility in King County, Washington. N Engl J Med. 2020; 382:2005–11. 10.1056/NEJMoa2005412 32220208 PMC7121761

[10] Garibaldi BT, Fiksel J, Muschelli J, Robinson ML, Rouhizadeh M, Perin J, Schumock G, Nagy P, Gray JH, Malapati H, Ghobadi-Krueger M, Niessen TM, Kim BS, et al. Patient trajectories among persons hospitalized for COVID-19: A cohort study. Ann Intern Med. 2021; 174:33–41. 10.7326/M20-3905 32960645 PMC7530643

[11] Bousquet G, Falgarone G, Deutsch D, Derolez S, Lopez-Sublet M, Goudot FX, Amari K, Uzunhan Y, Bouchaud O, Pamoukdjian F. ADL-dependency, D-Dimers, LDH and absence of anticoagulation are independently associated with one-month mortality in older inpatients with Covid-19. Aging (Albany NY). 2020; 12:11306–13. 10.18632/aging.103583 32576712 PMC7343508

[12] Michelozzi P, de’Donato F, Scortichini M, Pezzotti P, Stafoggia M, De Sario M, Costa G, Noccioli F, Riccardo F, Bella A, Demaria M, Rossi P, Brusaferro S, et al. Temporal dynamics in total excess mortality and COVID-19 deaths in Italian cities. BMC Public Health. 2020; 20:1238. 10.1186/s12889-020-09335-8 32795276 PMC7426899

[13] Zerah L, Baudouin É, Pépin M, Mary M, Krypciak S, Bianco C, Roux S, Gross A, Toméo C, Lemarié N, Dureau A, Bastiani S, Ketz F, et al, and APHP / Universities / Inserm COVID-19 research collaboration. Clinical characteristics and outcomes of 821 older patients with SARS-cov-2 infection admitted to acute care geriatric wards. J Gerontol A Biol Sci Med Sci. 2020. [Epub ahead of print]. 10.1093/gerona/glaa210 32845301 PMC7546043

[14] Wu Z, McGoogan JM. Characteristics of and important lessons from the coronavirus disease 2019 (COVID-19) outbreak in China: summary of a report of 72 314 cases from the Chinese center for disease control and prevention. JAMA. 2020; 323:1239–42. 10.1001/jama.2020.2648 32091533

[15] COVID-19 Point épidémiologique hebdomadaire du 4 juin 2020. Santé Publique France; 2020. https://www.santepubliquefrance.fr/content/download/257630/2628879

[16] Girvan G. 45% of COVID-19 Deaths in Nursing Homes & Assisted Living Facilities. Medium. 2020. https://freopp.org/the-covid-19-nursing-home-crisis-by-the-numbers-3a47433c3f70

[17] Marcon G, Tettamanti M, Capacci G, Fontanel G, Spanò M, Nobili A, Forloni G, Franceschi C. COVID-19 mortality in Lombardy: the vulnerability of the oldest old and the resilience of male centenarians. Aging (Albany NY). 2020; 12:15186–95. 10.18632/aging.103872 32788424 PMC7467374

[18] Lazarovici C, Somme D, Carrasco V, Baubeau D, Saint-Jean O. [Characteristics and resource use of emergency department users older than 75 years. Results from a French national study]. Presse Med. 2006; 35:1804–10. 10.1016/s0755-4982(06)74905-3 17159731

[19] Rochon PA, Gruneir A, Wu W, Gill SS, Bronskill SE, Seitz DP, Bell CM, Fischer HD, Stephenson AL, Wang X, Gershon AS, Anderson GM. Demographic characteristics and healthcare use of centenarians: a population-based cohort study. J Am Geriatr Soc. 2014; 62:86–93. 10.1111/jgs.12613 24383610

[20] Evans CJ, Ho Y, Daveson BA, Hall S, Higginson IJ, Gao W, and GUIDE_Care project. Place and cause of death in centenarians: a population-based observational study in England, 2001 to 2010. PLoS Med. 2014; 11:e1001653. 10.1371/journal.pmed.1001653 24892645 PMC4043499

[21] Liu W, Tao ZW, Wang L, Yuan ML, Liu K, Zhou L, Wei S, Deng Y, Liu J, Liu HG, Yang M, Hu Y. Analysis of factors associated with disease outcomes in hospitalized patients with 2019 novel coronavirus disease. Chin Med J (Engl). 2020; 133:1032–38. 10.1097/CM9.0000000000000775 32118640 PMC7147279

[22] Oksuzyan A, Juel K, Vaupel JW, Christensen K. Men: good health and high mortality. Sex differences in health and aging. Aging Clin Exp Res. 2008; 20:91–102. 10.1007/BF03324754 18431075 PMC3629373

[23] Robine JM, Cubaynes S. Worldwide demography of centenarians. Mech Ageing Dev. 2017; 165:59–67. 10.1016/j.mad.2017.03.004 28315698

[24] Franceschi C, Motta L, Valensin S, Rapisarda R, Franzone A, Berardelli M, Motta M, Monti D, Bonafè M, Ferrucci L, Deiana L, Pes GM, Carru C, et al. Do men and women follow different trajectories to reach extreme longevity? Italian Multicenter Study on Centenarians (IMUSCE). Aging (Milano). 2000; 12:77–84. 10.1007/BF03339894 10902049

[25] Gellert P, von Berenberg P, Oedekoven M, Klemt M, Zwillich C, Hörter S, Kuhlmey A, Dräger D. Centenarians differ in their comorbidity trends during the 6 years before death compared to individuals who died in their 80s or 90s. J Gerontol A Biol Sci Med Sci. 2018; 73:1357–62. 10.1093/gerona/glx136 29106492

[26] Jokanovic N, Tan EC, Dooley MJ, Kirkpatrick CM, Bell JS. Prevalence and factors associated with polypharmacy in long-term care facilities: a systematic review. J Am Med Dir Assoc. 2015; 16:535.e1–12. 10.1016/j.jamda.2015.03.003 25869992

[27] Yang J, Zheng Y, Gou X, Pu K, Chen Z, Guo Q, Ji R, Wang H, Wang Y, Zhou Y. Prevalence of comorbidities and its effects in patients infected with SARS-CoV-2: a systematic review and meta-analysis. Int J Infect Dis. 2020; 94:91–95. 10.1016/j.ijid.2020.03.017 32173574 PMC7194638

[28] Annweiler C, Guillaume S, Salles N, Aquino JP, Gautier J, Berrut G, Gurin O, Gavazzani G, on Behalf of the SFGG Covid-19 Study Group. National French Survey of Symptoms in People Aged 70 and Over Diagnosed with Covid-19. J Infec Deas. 2021; 72:490–94. 10.1093/cid/ciaa792. 32556328 PMC7337693

[29] Morandi A, Bellelli G. Delirium superimposed on dementia. Eur Geriatr Med. 2020; 11:53–62. 10.1007/s41999-019-00261-6 32297232

[30] Akbar AN, Gilroy DW. Aging immunity may exacerbate COVID-19. Science. 2020; 369:256–57. 10.1126/science.abb0762 32675364

[31] Franceschi C, Capri M, Monti D, Giunta S, Olivieri F, Sevini F, Panourgia MP, Invidia L, Celani L, Scurti M, Cevenini E, Castellani GC, Salvioli S. Inflammaging and anti-inflammaging: a systemic perspective on aging and longevity emerged from studies in humans. Mech Ageing Dev. 2007; 128:92–105. 10.1016/j.mad.2006.11.016 17116321

[32] Franceschi C, Bonafè M. Centenarians as a model for healthy aging. Biochem Soc Trans. 2003; 31:457–61. 10.1042/bst0310457 12653662

[33] Rosenberg ES, Dufort EM, Udo T, Wilberschied LA, Kumar J, Tesoriero J, Weinberg P, Kirkwood J, Muse A, DeHovitz J, Blog DS, Hutton B, Holtgrave DR, Zucker HA. Association of treatment with hydroxychloroquine or azithromycin with in-hospital mortality in patients with COVID-19 in New York state. JAMA. 2020; 323:2493–502. 10.1001/jama.2020.8630 32392282 PMC7215635

