# Supplementary material for: Centenarians in nursing homes during the COVID-19 pandemic
Source: Aging (Albany NY). 2021 Mar 2;13(5):6247–57. doi: 10.18632/aging.202743 (PMC7993710; doi:10.18632/aging.202743)
Supplement: Supplementary Table 1 [file aging-13-202743-s001.pdf]

**Supplementary Table 1. COVID-19 related symptoms, biology, treatment, and clinical outcomes in four different age groups (65-79 years vs. 80-89 years vs. 90-99 years vs. 100 years and over).**

| Characteristics | Patients 65 to 79 years old (n=57) | | Patients 80 to 89 years old (n=123) | | Patients 90 to 99 years old (n=129) | | Patients 100 and over (n=12) | | *p-value* |
|---|---|---|---|---|---|---|---|---|---|
| | n | % | n | % | n | % | n | % | |
| **Asymptomatic Symptoms** | 17 | 29.8 | 23 | 18.7 | 21 | 16.3 | 2 | 16.7 | 0.200 |
| Dyspnea | 15 | 26.3 | 41 | 33.3 | 59 | 45.7 | 4 | 33.3 | 0.051 |
| Dry cough | 11 | 19.3 | 30 | 24.4 | 43 | 33.3 | 2 | 16.7 | 0.165 |
| Fever | 21 | 36.8 | 54 | 43.9 | 56 | 43.4 | 6 | 50.0 | 0.767 |
| Hypothermia | - | - | 8 | 6.5 | 7 | 5.4 | 1 | 8.3 | 0.144 |
| Asthenia | 28 | 49.1 | 65 | 52.8 | 66 | 51.2 | 6 | 50.0 | 0.975 |
| Anorexia | 6 | 10.5 | 27 | 22.0 | 33 | 25.6 | 5 | 41.7 | **0.036** |
| Gastrointestinal signs | 10 | 17.5 | 15 | 12.2 | 31 | 24.0 | 2 | 16.7 | 0.103 |
| Ear, Nose, Throat (ENT) | 1 | 1.8 | 11 | 8.9 | 13 | 10.1 | 2 | 16.7 | 0.106 |
| Headache | 2 | 3.5 | 2 | 1.6 | 3 | 2.3 | - | - | 0.770 |
| Myalgia | 2 | 3.5 | 3 | 2.4 | 4 | 3.1 | - | - | 0.936 |
| Delirium | 6 | 10.5 | 14 | 11.4 | 22 | 17.1 | 2 | 16.7 | 0.465 |
| Worsening of depression | 4 | 7.0 | 5 | 4.1 | 8 | 6.2 | 3 | 25.0 | 0.074 |
| Altered consciousness | - | - | 8 | 6.5 | 10 | 7.8 | 1 | 8.3 | 0.111 |
| Fall | 4 | 7.0 | 11 | 8.9 | 11 | 3.4 | - | - | 0.918 |
| **Biological characteristics*** | | | | | | | | | |
| Anemia | 17 | 29.8 | 37 | 30.1 | 36 | 27.9 | 6 | 50.0 | 0.477 |
| Neutropenia | 6[a] | 14.3 | 6[b] | 6.4 | 4[c] | 3.8 | 1[d] | 10.0 | 0.113 |
| Lymphopenia | 13[a] | 31.0 | 29[b] | 30.9 | 27[c] | 25.2 | 4[d] | 40.0 | 0.633 |
| Thrombopenia | 1[a] | 2.4 | 10[b] | 10.6 | 10[c] | 9.3 | 1[d] | 10.0 | 0.365 |
| C-reactive protein>10mg/L | 27[f] | 73.0 | 63[g] | 70.8 | 73[h] | 72.3 | 7[e] | 77.8 | 0.994 |
| **Treatment** | | | | | | | | | |
| Antibiotics** | 29 | 50.9 | 79 | 64.2 | 93 | 72.1 | 10 | 83.3 | **0.024** |
| Azithromycin | 42 | 73.7 | 86 | 69.9 | 103 | 32.1 | 10 | 83.3 | 0.299 |
| Hydroxychloroquine | 22 | 38.6 | 40 | 32.5 | 37 | 28.7 | 1 | 8.3 | 0.186 |
| Hydration | 17 | 29.8 | 54 | 43.9 | 79 | 61.2 | 9 | 75.0 | **<0.001** |
| Preventive anticoagulation | 12 | 21.1 | 31 | 25.2 | 42 | 32.6 | 4 | 33.3 | 0.329 |
| Oxygen therapy | 13 | 22.8 | 48 | 39.0 | 55 | 42.6 | 5 | 41.7 | 0.065 |
| **Drug-drug interactions** | 35 | 61.4 | 53 | 43.1 | 51 | 39.5 | 5 | 41.7 | **0.046** |
| **Adverse event** | 3 | 5.3 | 10 | 8.1 | 20 | 15.5 | 1 | 8.3 | 0.129 |
| **Hospitalization in COVID unit** | 18 | 31.6 | 28 | 22.8 | 28 | 21.7 | 1 | 8.3 | 0.313 |
| **Home hospitalization** | 20 | 35.1 | 52 | 42.3 | 39 | 30.2 | 4 | 33.3 | 0.265 |
| **Palliative care** | 4 | 7.0 | 17 | 13.8 | 21 | 16.3 | 4 | 33.3 | 0.087 |
| **Death** | 8 | 14.0 | 24 | 19.5 | 34 | 26.4 | 6 | 50.0 | **0.029** |
| **Death due to COVID** | 5 | 83.3 | 17 | 28.8 | 23 | 76.7 | 6 | 100.0 | 0.099 |
| **Place of death** | | | | | | | | | |
| COVID unit | 7 | 12.3 | 12 | 9.8 | 8 | 6.2 | 1 | 8.3 | 0.304 |
| Nursing home | 1 | 1.8 | 12 | 9.8 | 26 | 20.2 | 5 | 41.7 | **<0.001** |

* Anemia was defined as hemoglobin level <120 g/L; Neutropenia as neutrophils level <1.5 G/L; Lymphopenia as lymphocytes level <1.0 G/L; Thrombopenia was defined as platelets level < 150 G/L.
**Antibiotics used were penicillin, ceftriaxone, macrolides (other than azithromycin) and tetracyclin.
Lower-case letters in superscript refer to the number of missing values: a=15; b=29; c=22; d=2; e=3; f=20; g=34; h=28.
